# Behcet's Disease and Endocrine System

**DOI:** 10.1155/2012/827815

**Published:** 2011-12-15

**Authors:** Onur Ozhan, Kerem Sezer

**Affiliations:** Division of Endocrinology and Metabolism, Department of Internal Medicine, School of Medicine, Mersin University, 33079 Mersin, Turkey

## Abstract

Behcet's disease (BD) is a chronic disease which is characterized by recurrent oral apthous ulcerations, recurrent genital ulcerations, skin eruptions, ocular involvements and other various systemic manifestations as well as systemic vasculitis. Endocrine involvement in BD regarding various systems can be seen. Hypophysis is one of the best and dense vascularized organs of the body, thus it is likely that it can be affected by BD. Not only anterior hypophysis functions, but posterior hypophysis functions as well can be affected. As BD is a disease of autoimmune process, it may be possible that adrenal insufficiency or alterations in the cortisol levels could be expected. Another concern is whether or not there is insulin resistance in patients with BD. The avaliable data suggests that there is an increased susceptibility to insulin resistance in patients with BD.

## 1. Introduction

Behcet's disease (BD) is a chronic disease which is characterized by recurrent oral aphthous ulcerations, recurrent genital ulcerations, skin eruptions, ocular involvements, and other various systemic manifestations as well as systemic vasculitis [1]. Endocrine involvement in BD regarding various systems can be seen. Here with this paper we have tried to review the literature in terms of BD “effects” on endocrine system.

## 2. Behcet's Disease and Hypophysis

Hypophysis is one of the best and densest vascularized organs of the body, thus it is likely that it can be affected by BD [2]. There are several studies regarding this fact in the literature. In one of these studies, Akdeniz et al. had studied the baseline and stimulated thyroid functions in BD. They recruited 30 patients consisting of 17 males and 13 females and 30 control subjects to their study. Thyroid function tests baseline and TSH levels at the 20th and 60th minutes after the administration of TRH were evaluated. In conclusion they stated that thyroid functions were normal in patients with BD; however, TRH stimulation was found to be decreased [3]. In another study where thyroid functions were evaluated, Aksu et al., found that thyroid function tests were within normal range in patients with BD; however, they did not assess the TRH-stimulated TSH levels [4]. Posterior hypophysis functions can also be affected in patients with BD which can represent itself with diabetes insipudus. Even though this entity is a rare feature of BD, there are several case reports being reported in the literature. In one of these, Khiari et al. reported that a 47-year-old man presented with oral and genital ulcers, skin lesions, and polyarthralgia. Two years after his first application he started to suffer from right uveitis and central diabetes insipidus without dysfunction of the anterior pituitary [5]. In another case report Szymajda et al., from USA, reported a 32-year-old man with diplopia and severe headaches who had transverse sinus thrombosis at the same time. They also stated that the patient was suffering from recurrent mouth and genital ulcers for more than 3 months before his application. During his hospitalization, the patient was reported having polyuria, polydipsia, hypernatremia, and hypotonic urine which were the stigmatas of diabetes insipidus. After the diagnosis, desmopressin was started and improvement in clinical status was achieved. As the mechanism of this pathology, vasculitis was accused [6]. Jin-No et al. [7], Otsuka et al. [8], and Hamza et al. [9], have studies also regarding the relationship between BD and diabetes insipidus.

 Another interesting question which has been a concern for recent years is: does prolactin levels have effect on the activation of BD? There are some studies available in the literature regarding this subject. In one of these studies Çil et al. found no significant difference in terms of hormone levels between the Behcet and control groups [10]. However in another study, Proençha et al. reported that serum prolactin levels were significantly higher in BD patients versus controls [11]. Another study by Atasoy et al. also had declared that patients with active BD had higher serum prolactin levels than the inactive and control groups. In their study prolactin levels in patients with active BD differed significantly from the healthy control subjects [12]. Nevertheless, a study which was conducted by Houman et al., found that the mean prolactin level in the BD group (mean = 13.76, SD = 6.82), was higher than the control group (mean = 10.13, SD = 5.46) with no statistically significant difference. Also the mean prolactin levels in all subgroups of patients with BD were higher than normal, but no statistically significant difference was shown between these subgroups [13]. The exact mechanisms of why prolactin is increased in BD remains unclear; however, there are some facts which has been suggested responsible. Prolactin has been shown to be an important modulator of cellular and humoral immunity which may play a role in the pathogenesis of several autoimmune diseases such as rheumatoid arthritis [14–16]. Increased prolactin levels and disease activity of systemic lupus erythematosus have been found to be associated with each other as well [17]. In this context, since BD is also a rheumatic disease of autoimmune basis, it might be suggested that prolactin levels could be elevated.

## 3. Behcet's Disease and Adrenal Glands

As BD is a disease of autoimmune process, it may be possible that adrenal insufficiency or alterations in the cortisol levels could be expected. Taking this fact into consideration Colak et al. had conducted a study investigating the effects of low- (1 microg; LDT) and standard- (250 microg; SDT) dose ACTH stimulation tests on adrenal cortex functions in patients with Behcet's disease; 18 patients with BD and 15 controls were recruited to this study. Both the patient and control groups were administered 1 microgram and 250 micrograms of ACTH, respectively, with 3-day intervals in between and responses of cortisol at the 0th, 30th, and 60th minutes after the administration of ACTH were evaluated. They found that there was no statistically significant difference between basal cortisol levels of BD and the control group; however, cortisol levels in the 60th minute of LDT were significantly low in patients with Behcet as compared to the control group. The peak cortisol responses to LTD were found to be decreased in Behcet's group as well. In this context they suggested that hypothalamopituitary axis is partially suppressed in BD [18]. A case report by Sánchez Sobrino et al., from Spain reports a longstanding Behcet's patient developing adrenal insufficiency, caused by isolated corticotropin deficiency. In this sense they reviewed the literature for the possible mechanisms of hypothalamic-pituitary injury in BD [19].

## 4. Behcet's Disease and Insulin Resistance

Another concern and studied subject is whether or not there is insulin resistance in patients with BD. In one of these studies Kim et al., evaluated this subject. They recruited 82 BD patients and 89 healthy controls to the study and measured the resistin and adiponectin levels at the time of enrolment along with the HOMA-IR evaluation with fasting plasma glucose and insulin levels. As their result they declared that there is an increased susceptibility to insulin resistance in patients with BD as compared to the controls and resistin could be an independent factor for this resistance [20]. In another study where peripheral insulin resistance was assessed with hyperinsulinemic-euglycaemic glucose clamp technique, Erdem et al., from Turkey, found that patients with BD exhibit peripheral insulin resistance which was thought to be because of inflammation and endothelial dysfunction [21].

## 5. Conclusion

With this review we have tried to have a look at what has been studied and evaluated in the literature regarding the relationship between BD and certain endocrine subjects via PubMed. As it may be seen, the available data is not too much. Since BD is a disease of autoimmunity and many diseases related with endocrinology have autoimmunity basis, we believe that further studies should be performed to understand if BD and at least some endocrine diseases interact with each other.

## References

[1] Sakane T, Takeno M, Suzuki N, Inaba G (1999). Behcet’s disease. *New England Journal of Medicine*.

[2] Aron DC, Finding CV, Thyrell JB, Greenspan FS, Gardner DG (2004). Hypothalamus and pituitary gland. *Basic and Clinical Endocrinology*.

[3] Akdeniz S, Colak S, Tuzcu AK, Bahceci M, Harman M (2006). Baseline and stimulated thyroid functions in Behcet’s disease. *Journal of the European Academy of Dermatology and Venereology*.

[4] Aksu K, Oksel F, Keser G (1999). Thyroid functions in Behcet's disease. *Clinical Endocrinology*.

[5] Khiari K, Cherif L, Hadj Ali I (2003). Central diabetes insipidus with Behcet disease. A case report. *Annales d'Endocrinologie*.

[6] Szymajda A, Eledrisi MS, Patel R, Chaljub G, Cepeda E, Kaushik P (2003). Diabetes insipidus as a consequence of neurologic involvement in Behcet’s syndrome. *Endocrine Practice*.

[7] Jin-No M, Fujii T, Jin-No Y, Kamiya Y, Okada M, Kawaguchi M (1999). Central Diabetes Insipidus with Behcet’s Disease. *Internal Medicine*.

[8] Otsuka F, Amano T, Ogura T, Ota Z (1995). Diabetes insipidus with Behcet’s disease. *Lancet*.

[9] Hamza M, Chamaki S, Boukris R (1989). Diabetes insipidus in a case of Behcet’s disease with neurologic manifestations. *Presse Medicale*.

[10] Çil AP, Karabulut AA, Koçak M (2010). Assessment of ovarian stromal artery Doppler characteristics and serum hormone levels in patients with Behçet disease. *Diagnostic and Interventional Radiology*.

[11] Proençha H, Ferreira C, Miranda M, Castanheira-Dinis A, Monteiro-Grillo M (2007). Serum prolactin levels and Behçet disease. *European Journal of Ophthalmology*.

[12] Atasoy M, Karatay S, Yildirim K, Kadi M, Erdem T, Senel K (2006). The relationship between serum prolactin levels and disease activity in patients with Behcet’s disease. *Cell Biochemistry and Function*.

[13] Houman H, Ben Ghorbel I, Lamloum M (2001). Prolactin levels in Behcet’s disease: no correlation with disease manifestations and activity. *Annales de Medecine Interne*.

[14] Reber PM (1993). Prolactin and immunomodulation. *American Journal of Medicine*.

[15] Neidhart M (1998). Prolactin in autoimmune diseases. *Proceedings of the Society for Experimental Biology and Medicine*.

[16] Chikanza IC (1999). Prolactin and neuroimmunomodulation: in vitro and in vivo observations. *Annals of the New York Academy of Sciences*.

[17] Jara LJ, Gomez-Sanchez C, Silveira LH, Martinez-Osuna P, Vasey FB, Espinoza LR (1992). Hyperprolactinemia in systemic lupus erythematosus: association with disease activity. *American Journal of the Medical Sciences*.

[18] Colak R, Ozkan Y, Cengiz SU, Saral Y, Kandi BC, Halifeoglu I (2006). A comparison between the effects of low (1 *μ*g) and standard dose (250 *μ*g) ACTH stimulation tests on adrenal cortex functions with Behçet's disease. *Journal of the European Academy of Dermatology and Venereology*.

[19] Sánchez Sobrino P, Fernández CP, Ferreiro JLL, Gil BM, Carballeira RP, García-Mayor RV (2009). Behçet disease with isolated ACTH deficiency. *Endocrinologia y Nutricion*.

[20] Kim SK, Choe JY, Park SH, Lee SW, Lee GH, Chung WT (2010). Increased insulin resistance and serum resistin in Korean patients with Behcet’s disease. *Archives of Medical Research*.

[21] Erdem H, Dinc A, Pay S, Simsek I, Turan M (2006). Peripheral insulin resitance in patients with Behçet’s disease. *Journal of the European Academy of Dermatology and Venereology*.

